# The function of the Norwegian municipal acute units fails to fulfill the intention of health authorities

**DOI:** 10.1080/02813432.2020.1717085

**Published:** 2020-01-24

**Authors:** Anne-Kari Johannessen, Sissel Steihaug

**Affiliations:** a Department of Nursing and Health Promotion, Faculty of Health Sciences, Oslo Metropolitan University, Norway;; b Health Services Research Unit, Akershus University Hospital, Lørenskog, Norway;; c Ekornveien 4. 0777 Oslo, Norway

**Keywords:** Municipal acute unit, municipal healthcare, collaboration, elderly, qualitative research

## Abstract

**Objective:** The aim of the study was to explore healthcare providers’ perceptions of how Norwegian municipal acute units (MAUs) possibly can reduce hospital admittance and improve service integration.

**Method and material:** Qualitative data were drawn from individual interviews with 40 healthcare providers, including general practitioners and staff in Norwegian MAUs, purchasing offices and home-based nursing services. Interview transcripts were analysed using systematic text condensation.

**Setting:** Two MAUs operated by 12 municipalities in eastern Norway.

**Results:** The healthcare providers disagreed on what MAUs are and should be. Frequent discussions between providers about which patients are appropriate for MAUs, as well as time- and resource-consuming procedures for patients’ admittance and discharge, have hampered the efficient operation of MAUs. Although, MAUs are operated by municipalities, the providers expressed that the units represent a new level of organisation with new boundaries for collaboration. Having many physicians in part-time positions and lacking physicians during night shifts were also characterised as problematic.

**Conclusion:** Several healthcare providers expressed uncertainty about the appropriateness of maintaining MAUs in Norway’s healthcare system, given their questionable capacity to meet Norwegians’ healthcare needs. It may appear that the MAUs are designed first to identify appropriate patients instead of identifying and mapping the population’s needs and, thereafter, designing optimal healthcare services.

**KEY POINTS**
- As of 2016, Municipal Acute Units (MAUs) are statutory healthcare services in Norway. Exploring patients’ and healthcare providers’ views on MAUs can improve the services.
- Healthcare providers disagreed on which patients were suitable for the units
- The units were perceived as a new (healthcare) level, entailing a new collaboration arena, with more bureaucracy and time expenditure
- The patients were satisfied with their treatment and care in the MAUs and the units’ proximity to their home

## Introduction

Ageing populations and the increased prevalence of chronic diseases pose major challenges for healthcare systems worldwide [1]. In response, many Western countries have reorganised their healthcare services in an effort to achieve more cost-effective solutions and relieve the burden placed upon hospitals [2–4]. The Norwegian healthcare system provides two tiers of care: primary care provided by general practitioners (GPs), home-based services, long-term care services and social care services; and secondary care provided by hospitals and specialists. GPs are paid in part by a capitation component depending on the number of patients on the list and partly on the basis of fee-for-service. The other health services are publicly financed and, largely, publicly provided.

As part of the Norwegian Coordination Reform launched in 2012, responsibilities were transferred from the central to the municipal governments [4]. Key objectives of the reform included reducing hospital beds, providing care for more patients through the primary healthcare system and ensuring more coordinated healthcare services. Several means have been implemented to achieve these ends. As a result of one initiative adopted in 2016, all Norwegian municipalities have been obliged to offer municipal acute beds in order to reduce hospitalisation, care for more patients in primary care and achieve more-integrated healthcare services [4,5].

This study examined two 24-h municipal acute units (MAUs), which are generally designed for patients diagnosed with conditions that can be managed with equipment less advanced than that maintained by hospitals [6,7]. The targeted population of MAUs includes patients ages 18 years or older who need medical observation and treatment in the short term (i.e. 3–5 days). The patients should have diagnosed, stable conditions; their needs might be due to exacerbations of symptoms of chronic diseases, infections requiring antibiotic treatment or conditions that call for pain relief [5]. As of 2017, there were 217 MAUs in Norway, and the national average number of patient beds filled was 40% [8]. Differing in size and services offered, MAUs are more commonly established in nursing homes, ‘houses of health’ in relation to a hospital or an emergency primary care centre, or as free-standing municipal or intermunicipal units [7]. At most MAUs, physicians are available only during the daytime, whereas an out-of-hours physician services the MAU at night [9].

Due to the lack of physicians present at night and uncertainty about the competence of MAU staff, GPs seem to be sceptical about MAUs and tend to prefer hospitalisation [10]. Leonardsen has reported that GPs found it challenging to decide which patients were appropriate for treatment in MAUs [11]. Moreover, the effect of maintaining MAUs in Norway has been found to be a mere 2% reduction in hospital admittance on average [12]. In MAUs co-located with an emergency primary care centre and with a physician present on a 24-h basis, the reduction in hospital admittance has been 5% on average. Municipalities that host intermunicipal units appear to have gained little from MAUs, while MAUs maintained by other organisations seem to have had no effect on reducing hospitalisation [12].

Because Norwegian health authorities continue working to establish MAUs in all of Norway’s municipalities, it is pivotal to know how MAUs realise the intentions of health authorities. Healthcare providers at the level of practice are key figures in MAUs, and thus, their experiences with and perceptions of MAUs are essential.

## Objective

The aim of the study was to explore healthcare providers’ perceptions of how Norwegian MAUs possibly can reduce hospital admittance and improve service integration.

## Setting

The study was conducted in two MAUs in eastern Norway and in eight of all 12 municipalities collaborating with MAUs. One of the MAUs opened in 2014, has 16 beds and represents collaboration among seven municipalities. The other MAU opened in 2016, has six beds, represents collaboration among five municipalities, and is also farther from the hospital. The cooperating hospital’s chief obligation is to provide 24-h telephone guidance on medical issues. The municipalities have reciprocal binding agreements regarding finances and admission and exclusion criteria.

The MAUs are staffed with nurses and physicians; the physicians do not work night shifts. The MAUs maintain an arrangement called the ‘diagnostic loop’, in which patients are sent to the hospital for rapid diagnostic clarification if the referring physician is unsure about whether admittance to the MAU is safe. In 2017, patients in the two MAUs studied were respectively 78- and 79-year old on average [13]; the number of patient beds filled was, respectively, 74% and 61%.

## Method

### Informants

Individual interviews were chosen for data collection. In close collaboration with the heads of the MAUs, emergency primary care centres, purchasing offices and home-based care services, the first author recruited provider informants representing four of the 12 collaborating municipalities: two large ones and two small ones. Using snowball sampling, the first author also recruited GPs and district medical officers from eight municipalities. In all, 40 healthcare providers were recruited, of whom 11 were management-level leaders; seven of these also performed clinical work. Physicians and healthcare providers from purchasing offices and home-based nursing services were chosen based on whether they had prior experience collaborating with MAUs. The providers interviewed are listed in Table 1.

**Table 1.** Healthcare providers interviewed.

Workplace/profession	No	M	F	Notes
Municipal Acute Unit:				
Nurse	9		9	7 from one MAU and 5 from the other MAU
Physician	3^a^	3		
Purchasing office:				
Nurse	6		6	Represents 4 municipalities
Social worker	2		2	
Social educator	2	1	1	
Home-based care:				Represents 4 municipalities
Nurse	8	1	7	
Physicians:				
General practitioner	6^b^	3	3	Represents 8 municipalities
District medical officer	3	3		
Out-off-hours-doctor	1		1	
Total	40	11	29	

^a^One physician had full time position on MAU, 1 had combined position between MAU and Emergency clinic and 1 had combined position between MAU, Nursing home and Emergency clinic.

^b^Four physicians had full time positions as GPs and *Interviews.*

The interviews were semi-structured and followed interview guides developed in advance. During interviews, informants were asked about the function of the MAUs, the competence of their staff, their targeted patient groups, their collaboration with primary healthcare providers and whether they operated as part of clinical pathways. The interviews were conducted from March to December 2017. Each interview lasted approximately 45 min, was recorded on digital recording equipment and was transcribed verbatim by the first author.

Both authors (i.e. a nurse and a physician) were acquainted with Norwegian intermediate units from earlier research and had particular expectations regarding what they would find, for example, challenging collaboration about the identification of patients appropriate for the units. The interviewer was especially conscious of her potential preconseptions and sought to counteract that by seeking new and contrasting views.

### Analysis

Both authors analysed the interview transcripts by employing systematic text condensation, a four-step method developed by Malterud [14]. The first step involved reading all the material to obtain an overall impression and to identify preliminary themes. In the second step, we identified meaning units—that is, sections of text representing different aspects of the preliminary themes developed during the first step—and coded them under different headings, for instance, ‘Physicians struggled with referring patients’ and ‘Time-consuming collaboration at discharge’. In the coding, we focussed on the MAUs’ functions and how the units might realise the intentions of health authorities. The meaning units were repeatedly sorted into code groups and shifted from one group to another until each was placed under an appropriate heading. Code groups could be merged or divided, and we ended up with three groups. The meaning units in the group ‘Physicians struggled with referring patients’ and ‘Time-consuming collaboration at discharge’ were, for example, gathered and placed under the new heading ‘Problematic collaboration at admission and discharge’. The third step involved establishing subgroups exemplifying vital aspects of each code group and analysing each subgroup separately. We then condensed the content of each code group and selected quotations that we thought appropriately illustrated the essence of the descriptions. In the fourth and final step, we synthesised the condensates from each code group to form a generalised description that reflected the major findings. Each code group was given an appropriate heading. Our example, ‘Problematic collaboration on admission and discharge’ was given the final heading ‘Lack of collaboration and cumbersome routines obstruct the use of MAUs’. The generalised descriptions of the condensates from the three code groups constitute our results and are presented in the Results section.

### Ethics

Written informed consent was obtained from the informants before data collection. Informants were assured that their identities and collected data would be kept confidential and that they could withdraw from the study at any time. The study followed the Declaration of Helsinki on ethical principles for medical research involving human subjects. The Regional Committee for Medical and Health Research Ethics concluded that the study was not regulated by the Health Research Act (2016/2277/REK sør-øst A). Local privacy-protection advisors at Akershus University Hospital HF (ref 17-058) approved the study.

## Results

### Different opinions about what the MAUs are and what they should be

Depending on their occupational backgrounds and positions within the municipal healthcare system, the providers had different opinions about what kinds of services the MAUs represented and how they should be developed. Physicians and nurses in the MAUs maintained that the MAU admittance criteria were adequate and clear, and that the targeted patient group was realistic. The collaborating partners, by contrast, found the criteria to be too narrow and rigid and were of the opinion that more patients without clarified diagnoses should be accepted. Referring physicians and providers in purchasing offices expressed that the number of patients in the MAUs’ target group was quite limited in practice and that GPs strived to find them. Some providers in the MAUs and purchasing offices thought that the two MAUs had found their niche but needed to widen their admission criteria. Other informants, especially GPs and some purchasers, were more sceptical, however. A purchaser in a small non-host municipality put it this way:

I think that the MAU is not the service that we need most of all; we spend a lot of money on it. The patients who had a stay in an MAU had problems that we could have managed ourselves. We would have profited by establishing beds locally—for example, in the nursing home.

Both MAUs have received patients from outside the target group and experienced that patients’ stays were too long. Providers in the smaller unit said that the patients were generally appropriate but that several needed longer stays than had been stipulated. Both physicians and nurses in the larger MAU in a host municipality, however, reported having longer patients stays because they often felt forced to fill free beds and accept patients who needed care when beds at the nursing homes were filled and home-based services were overburdened. They complained about the unit’s development in the direction of a nursing home, and several nurses described their jobs as being boring and lacking professionally stimulating tasks. A physician in this MAU said:

I really fear that the MAU will end up becoming a nursing home if it doesn’t change its profile. To counteract that trend, we should consider offering beds to patients who are nearly ready to be discharged from the hospital instead of letting patients who need nursing home care occupy our beds.

All of the informants emphasised that a constant shortage of beds at nursing homes challenged the operation of the MAUs and that the units were often (mis)used as nursing homes. They described the pressure placed upon MAUs to accept patients in need of care as a result of health authorities’ strenuous efforts to expand home-based services at the expense of beds at the institutions. Providers in purchasing offices and in home-based services reported cases of severely ill patients who were treated at home. Very few informants agreed with the trend of reducing the number of beds at institutions and all but one, a nurse leader, thought that nursing homes had far too few temporary beds. When asked where the patients had been when not in the MAU, most informants answered, ‘In hospital’, whereas some said, ‘In a nursing home’. Few meant that the patients had been at home.

### Competence and localisation are important for the MAUs’ function

Most of the MAUs’ collaborating partners believed that staff in both MAUs generally provide good services to patients, even though some questioned whether their competence was sufficient and the diagnostic equipment adequate. One MAU physician claimed that not all physicians in the MAU had adequate competence in general practice. Both physicians and nurses employed in the two MAUs perceived that the absence of a physician in the MAUs at night was problematic. The nurses described the situation as incredibly stressful, and some admitted contemplating taking sick leave to avoid working night shifts. The diagnostic loop was also attributed to the lack of physicians on night shifts. Some providers found the loop to be important for ensuring patients’ safety at night, whereas others perceived the arrangement to be part of a bureaucratic system that burdens the hospital with additional work and frustrates patients who experience long wait times in the hospital’s emergency ward.

Several physicians and staff in purchasing offices pointed out that the MAUs’ co-localisation with emergency primary care centres would have been favourable for better patient flows, for improved interprofessional collaboration and for admitting appropriate patients to the units. Several purchasers and many GPs argued that MAUs should be organised and operated as part of existing nursing homes. A GP in a small non-host municipality expressed this view:

Instead of having separate MAUs, we should establish MAU beds in nursing homes and operate the MAUs together with the nursing homes. Such an arrangement implies flexibility in the use of beds and healthcare professionals and the more efficient operation of municipal healthcare service as a whole.

Many informants stated that the MAUs were expensive for small municipalities, largely because they used the units less than larger municipalities did, and several wanted to withdraw from the MAU collaboration. The larger MAU’s proximity to the hospital was a reason stated for using the hospital more often than the MAU.

### Lack of collaboration and cumbersome routines obstruct the use of MAUs

The informants described collaboration as challenging between the MAUs and their collaborating partners, especially in regard to patient admittance and discharge from the units. Referring physicians, especially GPs, found it demanding to have patients admitted to the MAUs and claimed that both MAUs observed strict, categorical admission criteria. They also characterised the application procedure as difficult and taking from 30 min to an hour, whereas hospital admittance could be processed in 10 min. MAU physicians were described as demanding a great deal of information that was difficult to obtain during home visits, and the GPs reported cases requiring several phone calls back and forth. Many GPs said they had stopped referring patients to the MAUs altogether. A GP working in a large municipality provided an example of challenging collaboration:

Some days ago, I made a home visit to an older physically impaired woman with a fever who seemed to be a little demented. I expected a certain accommodating attitude from my colleague at the MAU when I requested her admission. Instead, I was asked several critical, detailed questions that I couldn’t answer quickly. I felt as if I was not believed or accounted for in relation to my colleague, like a schoolboy in front of his teacher. Moreover, the MAU physician probably had less clinical experience than me.

Different reporting routines and computer systems between the purchasing offices that finance and run the home-based services and the MAUs hampered their collaboration. MAU employees thought that the purchasing offices often made unfair demands by continually requesting revised care plans when patients were about to be discharged. Moreover, nurses described the documentation process as time-consuming. On the other hand, the staff at purchasing offices reported that the MAUs’ application procedure for patients’ discharge was unstructured compared to that of hospitals. Purchasers viewed the fact that MAU providers have promised patients primary care services during their stay at the unit as problematic. A purchaser in a small, non-host municipality stated:

It’s very annoying when the MAU promises services to patients on our behalf. We have to have an unpleasant conversation and apologise that we don’t have the capacity to fulfil those promises. The loss of the patient’s confidence is often the result.

## Discussion

### The study’s strengths and weaknesses

For data collection, we have used qualitative interviews that followed interview guides developed in advance. Individual interviews were chosen instead of group interviews because of the difficulty of assembling healthcare professionals for group meetings. Group interviews could probably have provided more information based on discussions and participants maintaining different opinions. We have also experienced, however, that dominating participants have constrained other participants from offering their opinions in group interviews. Our informants were purposively sampled to ensure a variety of professionals and a wide range of practitioners. The 40 professionals represented a range of occupations and positions. Because some were leaders and many providers worked at the level of practice, they were able to provide varied and multifaceted information and perspectives. The first author is an experienced interviewer, and both authors have expertise in the field by virtue of their respective professional backgrounds as a nurse and a GP. Being conscious of our preconceptions about the role of intermediate units in the Norwegian healthcare system formed during earlier research, for example, challenging collaboration between the units and their collaborating partners, encouraged a search for information that would counter our preconceptions. Discussing the research process and the results with several other researchers with different backgrounds and opinions challenged our viewpoints and interpretations. A study’s internal validity indicates whether the study actually investigated what it aimed to investigate and whether it was conducted according to robust scientific methods. We have aimed at describing the conducting of the study in a way that allows the reader to follow the research process and evaluate the study’s internal validity. The study was conducted in a relatively densely populated area of eastern Norway, and the results may not be applicable to more rural areas. Nevertheless, the results correspond to the findings of other studies of MAUs, and the providers’ accounts reflect current clinical practice. We argue that the discussion about which patients are appropriate for MAUs, the limited availability of institutional beds, and MAUs as a new organisational level that poses new problems for collaboration are factors of relevance to other municipalities in Norway and beyond.

### Is the concept of MAUs an improvement for the Norwegian healthcare system?

Depending on their individual occupational backgrounds and current positions, the providers expressed different opinions about the function of MAUs in the Norwegian healthcare system. MAU providers thought that the units had found their niche and function according to the intentions of health authorities. Many collaborating partners, however, found the MAUs’ admission criteria to be overly narrow and expressed that patients described as being in the target group hardly exist in practice. Most informants appraised the competence of MAU staff as sufficient and the bed capacity as adequate, provided that patients’ stays in the units were not too long. However, problematic collaboration regarding patients’ admittance and discharge compromised the MAUs’ expediency. Swanson et al. have shown that MAUs rarely comply with Norwegian health authorities’ aim to reduce hospital admittance [12]. Below, we discuss healthcare providers’ perceptions of how MAUs possibly can reduce hospital admittance and improve service integration.

### Do MAUs relieve hospitals?

Many informants considered the MAUs as primarily extensions of hospitals and supposed that, if no MAU existed, then the patient would have been in hospital. This suggests that MAUs relieve hospitals. However, others argued that the MAUs primarily relieve purchasing offices and home-based care services, which often desperately seek beds at institutions. Staff at the MAUs reported feeling constrained frequently by having to care for patients in need of nursing home care at the expense of patients who need more active treatment. In many instances, the providers discussed whether fragile elderly patients with complex conditions should be treated in MAUs, and the fact that they were, especially in the larger MAU, implies that the units also relieved nursing homes. Many GPs found it too challenging and time-consuming to refer patients to the MAUs, and some had stopped referring them altogether, which suggests that the relief provided to hospitals has been less than it could be. Studies of similar units have also shown that GPs were unsure about whether MAU employees had sufficient competence, and as a result, they preferred to refer their patients for hospitalisation [8,10,11].

The absence of physicians at the MAUs at night caused undue stress for nurses, with the potential consequence that patients deemed too ill could be refused admittance. The need for the diagnostic loop to safeguard patients may indicate that staffing at the MAUs is not optimal for the relevant patient group and, in turn, does not effectively relieve hospitals. This is according to Swanson et al.’s finding that having a physician on duty around the clock was a prerequisite for MAUs’ 5% reduction in hospital admittance [12]. Many informants from the larger MAU and their collaborating partners pointed out that the MAU’s proximity to the hospital increased the likelihood that patients would be referred to the hospital instead of to the MAU.

### Do MAUs promote more integrated healthcare services?

The MAUs’ potential contribution to help Norway achieve more integrated healthcare services presumes collaboration. Results from the study reveal that collaboration between the MAUs and their collaborating partners has been challenging, primarily due to disagreements about which patients are medically appropriate for the units and problems in connection with patients’ admission and discharge. When they perform home visits, GPs often need to find institutional beds for patients and, as Leonardsen also found [11], to focus on where to refer patients and which ones could benefit from a stay in the MAU. MAU providers, however, were more focussed on which patients would be the best fit for the unit. Providers in purchasing offices and home-based services wanted patients to stay in the MAUs for as long as possible in order to relieve home-based services. That such conflicting interests because of different commitments and goals can result in challenging collaboration aligns with a study addressing an intermediate unit [15]. That informants disagreed about the inclusion criteria and several primary care providers expressed that the target patient group hardly exists may be related to the MAU as a new concept framed for particular diagnoses defined in advance [5]. It appears that, in practice, the inclusion criteria do not meet the needs identified. Different perceptions of care plans as well as different reporting routines and computer systems were identified as making collaboration between MAU nurses and staff in purchasing offices challenging.

The significance of good relationships between providers, knowledge about and mutual respect for each other’s work and competence, and sufficient time and resources for ongoing relationship building has been emphasised in the literature [16,17]. A collaborative forum of providers at the practical level instead of leaders, therefore, would likely contribute to improved cooperation and patient flow. New initiatives introduced by health authorities may have unintended consequences for clinical practice. The MAUs constitute parts of the municipal healthcare system but function as autonomous units that bring with them an additional administrative level. That new arena for cooperation has resulted in all parties involved having had to expend considerable time and energy on negotiations that do not seem to have improved the integration of services. Several informants stressed that MAUs should be located in connection to emergency rooms or nursing homes, which would likely improve patient flow.

### Are there sufficient beds for patients in need?

Limited bed capacities in municipal healthcare services align with health authorities’ goal to reorganise Norwegian healthcare services. The Coordination Reform highlights the importance of allowing patients to remain at home for as long as possible and reducing admissions to both nursing homes and hospitals [4]. Such emphasis implies that the threshold for granting municipal healthcare services has been noticeably higher and that patients treated in primary care have more serious, complex and treatment-intensive conditions than before the reform was introduced [18], which informants corroborated. Although, a few informants characterised that development as appropriate, far more thought that the development has gone too far. All but one informant emphasised the lack of temporary beds at nursing homes and that, despite intentions, the MAU was used instead of nursing homes to a certain extent. Informants exerting a great deal of time and energy on negotiations may indicate that beds in institutions for patients who need them are simply too few.

Some GPs argued that MAUs as part of local nursing homes could allow a more flexible use of resources. In these MAUs, employees use patients’ GPs as consultants to a great extent [19]. Some informants wished that MAUs were located close to an emergency primary care centre in order to safeguard patients at night. Swanson and Hagen [20] found that MAUs in combination with emergency primary care centres and with physicians present 24 h resulted in fewer acute hospital admissions.

## Conclusion

The study revealed that several healthcare providers remain unsure about MAUs’ appropriateness in the Norwegian healthcare system. It seems that most patients do not meet the criteria for MAU admission. All but one informant underscored the considerable lack of temporary beds in nursing homes and the frequent use of MAUs instead of nursing homes. Despite their municipal ownership, MAUs constitute a new level of organisation in terms of cooperation among healthcare providers. Frequent discussions among providers about which patients are appropriate for the units, as well as time- and resource-consuming procedures concerning patients’ admittance and discharge, have hampered the efficient operation of the units. This predicament raises the question of whether MAU services are the best solution for Norway’s current healthcare needs. It may appear that the MAUs were designed first for subsequently to find the appropriate patients instead of mapping and identifying the population’s needs and, consequently, designing optimal healthcare services based on these needs.

## Implications

Norwegian municipalities are obliged to offer municipal acute beds. Before establishing a new service as a part of the healthcare system, it is important for every municipality to identify the target group’s needs, the most appropriate organisational structure and to invest considerable effort on measures designed to strengthen relational and structural collaboration.

## References

[1] Kuhlmann E, Annandale E. Researching transformations in healthcare services and policy in international perspective: an introduction. Curr Sociol. 2012;60(4):401–414.

[2] Vabø M. Norwegian home care in transition – heading for accountability, off-loading responsibilities. Health Soc Care Community. 2012;20(3):283–291. 22356449 10.1111/j.1365-2524.2012.01058.x

[3] Lappegard O, Hjortdahl P. Perceived quality of an alternative to acute hospitalization: an analytical study at a community hospital in Hallingdal, Norway. Soc Sci Med. 2014;119:27–35. 25137645 10.1016/j.socscimed.2014.08.014

[4] Meld. St. 47 2008-2009. Samhandlingsreformen. Rett behandling-på rett sted-til rett tid [[Report No 47 (2008-2009). The Coordination Reform. Proper treatment-at the right place and right time]. Oslo: Helse- og omsorgsdepartementet [Norwegian Ministry of Health and Care Services. English summary of the White paper. Available from: https://www.regjeringen.no/en/dokumenter/report.no.-47-to-the-storting-2008-2009/id567201/

[5] Den Norske L. Medisinskfaglig veileder for kommunale akuttplasser (KAD)[The Norwegian Medical Association. Medical advisor for municipal emergency wards (KAD)]. 2014. Available from: https://legeforeningen.no/PageFiles/176624/140507%20KAD%20veileder.pdf

[6] Helsedirektoratet. Kommunenes plikt øyeblikkelig hjelp døgnopphold 2016. [The Norwegian Directorate of Health. The municipalities’ duty on immediately help the 24-hour stay in 2016. Available from: https://helsedirektoratet.no/publikasjoner/kommunenes-plikt-til-oyeblikkelig-hjelp-dognopphold-veiledningsmateriell

[7] Skinner MS. Døgnåpne kommunale akuttenheter: en helsetjeneste-modell med rom for lokale organisasjonstilpasninger [Municipal acute bed units: a national health service model with scope for local adaptation. Tidsskrift for Omsorgsforskning. 2015;1:131–144.

[8] Helsedirektoratet. Status for øyeblikkelig hjelp 2017 for kommunalt døgntilbud. [The Norwegian Directorate of Health. Status for immediate help 2017 for municipal care around the clock]. 2018. Available from: https://helsedirektoratet.no/Documents/Nyheter/Status%20for%20kommunalt%20døgntilbud%20for%20øyeblikkelig%20hjelp%202017.pdf

[9] Grimsmo A, Løhre A. Erfaringer med etablering av kommunalt øyeblikkelig hjelp døgntilbud. [Experiences with the establishment of municipal acute wards. Utposten. 2014;43:14–17.

[10] Skinner MS. Skeptiske leger og tomme senger? Bruken av de kommunale akutte døgnplassene [Skeptical doctors and empty beds? The use of the municipal emergency rooms]. Senter for Omsorgsforsknings- Rapportserie nr 10/2015. Available from: https://brage.bibsys.no/xmlui/bitstream/handle/11250/2365378/rapport_10_2015_tomme%20senger_web.pdf?sequence=1&isAllowed=y

[11] Leonardsen A-C, Del Busso L, Grøndahl VA, et al. General practitioners’ perspectives on referring patients to decentralized acute health care. FAMPRJ. 2016;33(6):709–714. 10.1093/fampra/cmw087 27543796

[12] Swanson J, Alexandersen N, Hagen TP. Kommunale akutte døgnenheter, legeberedskap og avstander. [Municipal acute units, medical emergency, and distances]. Rapport, Avdeling for helseøkonomi, Universitetet i Oslo; 2015. Available from: https://www.regjeringen.no/contentassets/477c27aa89d645e09ece350eaf93fedf/no/sved/08.pdf

[13] Korvann G. Etablering av KAD bidrar til færre sykehusinnleggelser og gode pasientforløp- upublisert [Establishing MAU contributes to fewer hospital admissions and good patient care - unpublished]. Oslo; 2018.

[14] Malterud K. Systematic text condensation: a strategy for qualitative analysis. Scand J Public Health. 2012;40(8):795–805. 23221918 10.1177/1403494812465030

[15] Johannessen A-K, Luras H, Steihaug S. The role of an intermediate unit in a clinical pathway. Int J Integr Care. 2013;13(1):e012. 23687484 10.5334/ijic.859 PMC3653277

[16] Tsasis P, Evans JM, Owen S. Reframing the challenges to integrated care: a complex-adaptive systems perspective. Int J Integr Care. 2012;12(5):e190. 23593051 10.5334/ijic.843 PMC3601537

[17] Nancarrow SA, Booth A, Ariss S, et al. Ten principles of good interdisciplinary team work. Hum Resour Health. 2013;11(1):19. 23663329 10.1186/1478-4491-11-19 PMC3662612

[18] Haukelien H, Vika H, Vardheim I. Samhandlingsreformens konsekvenser i de kommunale helse og omsorgstjenestene: sykepleieres erfaringer [The consequences of the cooperation reform in the municipal health and care services: nurses’ experiences]. Bø i Telemark: Telemarksforskning; 2015. Available from: https://www.nsf.no/Content/2621976/Samhandlingsreformens%20konsekvenser%2017%2008.pdf

[19] Lappegard O, Hjortdahl P. The choice of alternatives to acute hospitalization: a descriptive study from Hallingdal, Norway. BMC Fam Pract. 2013;14(1):87. 23800090 10.1186/1471-2296-14-87 PMC3698089

[20] Swanson JO, Hagen TP. Reinventing the community hospital: a retrospective population-based cohort study of a natural experiment using register data. BMJ Open. 2016;6(12):e012892. 10.1136/bmjopen-2016-012892 PMC5168673 27974368

